# A 12-month follow-up of primary and secondary root canal treatment in teeth obturated with a hydraulic sealer

**DOI:** 10.1007/s00784-020-03590-0

**Published:** 2020-09-28

**Authors:** Giulia Bardini, Laura Casula, Emanuele Ambu, Davide Musu, Montse Mercadè, Elisabetta Cotti

**Affiliations:** 1 Department of Conservative Dentistry and Endodontics, University of Cagliari, Cagliari, Italy; 2 Department of Medicine and Public Health, University of Cagliari, Cagliari, Italy; 3 Department of Dentistry, University of Barcelona, Barcelona, Spain

**Keywords:** Root canal obturation, Bioactive sealers, Single cone, Endodontic outcome

## Abstract

**Objectives:**

This randomized, controlled, pilot study assessed the outcome of non-surgical primary/secondary root canal treatments either with a novel bioactive sealer and the single-cone technique or with gutta-percha, zinc oxide-eugenol sealer (ZOE), and warm vertical compaction.

**Materials and methods:**

Sixty-nine patients were randomly divided into two groups that were treated using the single-cone technique with BioRoot^TM^ RCS (Septodont) (BIO group) or warm vertical compaction with gutta-percha and ZOE sealer (PCS group). Two subsamples (BIOAP and PCSAP) comprised the cases with apical periodontitis. Treatment was undertaken by four residents using a standardized instrumentation and disinfection protocol. The periapical index (PAI) was recorded, and clinical and radiographic follow-up performed at 1, 3, 6, and 12 months. Treatment success was assessed according to “periapical healing” and “tooth survival”. The test for the equality of proportions, *t* tests for the equality of means, and non-parametric K-sample tests for the equality of medians were applied when appropriate.

**Results:**

The survival rate was similar in the BIO and PCS (*p* = 0.4074) and the BIOAP and PCSAP groups (*p* = 0.9114). The success rate was higher in the BIO groups, but not statistically significant (*p* = 0.0735). In both BIOAP and PCSAP groups, a progressive decrease in the PAI was observed.

**Conclusion:**

At 12 months, both techniques showed reliable results. Further studies and longer follow-ups are needed.

**Clinical relevance:**

This study documents the feasibility of using a bioactive sealer in conjunction with the single-cone technique to obturate the root canal and obtaining a predictable outcome.

**Trial registration:**

ClinicalTrials.gov Identifie: NCT04249206

## Introduction

Apical periodontitis (AP) is a chronic inflammatory disease caused by an endodontic infection and is characterized by hard tissue resorption and destruction of periapical tissues [1, 2]. Apical periodontitis can be prevented or treated by an appropriate root canal treatment (RCT) [3]. According to the recent literature, the estimated weighted success rates of primary and secondary RCTs range between 68–85% and 70–86%, respectively, when strict criteria are used [3–7]. The quality of the root canal filling is a very important potential prognostic factor influencing the success of RCTs [6]. A state-of-the-art endodontic obturation obtained using gutta-percha (GP) cones together with a sealer adapted to the canal walls should prevent microorganisms and fluids from passing through the canal to the apical tissues by sealing the entire system [8, 9].

In recent years, bioactive endodontic cements (*Portland’s bioceramics*) have been introduced to the market; they have been used as pulp capping agents, as filling materials to seal endodontic/periodontal joins or, more recently, as sealers to be used in conjunction with GP [10]. The precursor of these cements, mineral trioxide aggregate (MTA), which is a Portland-derived cement, exhibited excellent hydraulic properties (since it sets and seals well in the presence of moisture because its properties are enhanced by the interaction with tissue fluids), biocompatibility, and induction of hard tissue formation [11, 12]. Subsequently, developed products have different compositions, most of which contain calcium and silicate [10, 13–17], and show similar performances [18–20]. A common property of these cements is their “bioactivity”; when they are in contact with tissue fluids, they release calcium ions and produce calcium hydroxide and apatite on their surfaces with the potential to create an interfacial layer between the sealer and the dentin walls [11, 12, 21]. Researchers have also reported a decreased inflammatory response in bone in the presence of these products [22–25]. BioRoot^TM^ RCS (Septodont, Saint-Maur-de-Fossés, France) is a tricalcium silicate–based bioactive cement developed to be used as a sealer together with GP points and the single-cone technique or with cold lateral condensation for permanent root canal obturation [26, 27]. There is a paucity of information in the specialized literature on the outcomes achieved with the new bioactive sealers used with the single-cone obturation technique in vivo; the latter (sealers and technique) are often advertised.

The purpose of this *pilot* study was to evaluate the outcome of endodontic primary and secondary treatment of teeth prepared with a similar protocol and obturated using either the single-cone technique with gutta-percha and a bioactive sealer or warm vertical compaction with gutta-percha and a ZOE sealer at a 1-year follow-up. This is intended to be the first part of a modular project that will provide data on the behaviour of the two groups over time (at 1 year, 2 years, and 4 years).

## Materials and methods

The pilot study was approved by the Ethics Committee (PROT. PG/2017/16759, Ca, November 2017) and was conducted in the Department of Conservative Dentistry and Endodontics of the University Hospital in accordance with the Declaration of Helsinki of 1975 (as revised in 2000) between 1 May 2016 and 31 December 2017.

### Inception cohort

Fifty-five Caucasian subjects aged between 18 and 80 years who were referred for endodontic treatment at the university clinic were included in the study. The selected subjects had at least one permanent single or multi-rooted mature tooth with signs and/or symptoms indicating the need for endodontic treatment (primary or secondary) according to ESE guidelines [9], and teeth requiring retreatment with a poor prognosis (root canal morphology altered) were excluded. Clinical and medical data were recorded before treatment. During the visit, each tooth was clinically examined to determine whether it needed endodontic treatment; the history of pain was assessed, and responses to sensitivity tests, palpation, and percussion were performed [28]. One or more periapical radiographs of the involved teeth were obtained at baseline and evaluated to assess the crown, root, and periapical status.

Patients with any medical condition, immune-compromised status, or with an overall poor prognosis for their treatment were not included.

Informed consent to undergo the treatment and follow-up and a second consent to participate in the study were obtained from all patients before treatment commenced.

The 55 selected patients required root canal treatment of 84 single or multi-rooted teeth.

Endodontic therapy was performed using a standardized protocol that varied only in terms of the technique and sealer used for the obturation of the root canal. Four endodontic residents performed the primary and secondary RCTs, who were divided into two groups of two (depending on the day of the week they worked in the hospital). The clinical supervisor of the day assigned each resident either Bioactive cement or ZOE sealer respectively. This was randomly done by a flip of the coin. Every day, each patient was randomly assigned to one technique or another: the first patient who arrived on the same day of treatment was randomly assigned (by a flip of the coin) to one of the two obturation groups, while the second was assigned to the other group; the third patient was assigned to the initial group, and so on until the end of the day.

### Dental treatment and follow-up

Local anaesthesia was administered, the teeth were isolated under a rubber dam, and the root canals were subjected to preflaring using K-files 08/10/15 (Kerr© Corporation, Orange, California) and NiTi Protaper Next^TM^ X1, X2, and X3 rotary files (Dentsply Sirona, Ballaigues, Switzerland) when necessary. For the secondary RCTs, the GP and sealer were removed by hand and with rotary instrumentation by using Gates-Glidden drills #4, #3, and #2 (in this sequence) and 0.1 mL of solvent (Endosolv® E, Septodont, Saint-Maur-des-Fossés, France); the canals were then renegotiated by hand with K-files. In all cases, the working length was assessed with the apex locator (DentalPort ZX, J. Morita MFG. CORP©, Kyoto, Japan) and confirmed with one or more periapical radiographs Kodak ultra-speed dental film, size 31 × 41 mm (Carestream Health©, Stuttgart, Germany) and X-safe 70 70 KV/8 mA (Cefla Medical Equipment, Imola, Italy). The root canals were continuously irrigated with 5% sodium hypochlorite (Niclor 5-Dentale, Ogna lab, Muggiò, Italy) throughout the instrumentation.

Following instrumentation, the canals were dried with sterile paper points and obturated as follows:

- BIO group: A standardized GP master cone that fit snugly at the working length was selected; BioRoot^TM^ RCS (Septodont) was prepared according to the manufacturer’s instructions. A coating of the sealer was applied onto the canal walls using the GP point. Obturation was completed by inserting the GP master cone that had been previously coated with the sealer into the canal; a hot instrument was used to remove the excess GP.
- PCS group: Warm vertical compaction with GP and Pulp Canal Sealer^TM^ EWT (Kerr© Corporation, Orange, CA) was performed [29].

A periapical radiograph was obtained to assess the quality of the root canal fillings, and subsequently, all teeth were restored with direct composite.

Clinical and radiographic follow-up were performed at 1, 3, 6, and 12 months for each tooth, and data were recorded in a dedicated chart and updated at every follow-up.

All radiographs were digitally scanned, saved in JPEG format and imported into ImageJ software version 1.41 (National Institute of Health, Bethesda, MD). The application Turbo Reg (Biomedical Imaging Group, Lausanne, Switzerland) was used to reduce the distortion factors of the radiographs [30].

Two trained and calibrated examiners (weighted kappa values, *k* = 0.8 for inter-examiner agreement and *k* = 0.9 for intra-examiner agreement) [31] assigned a PAI score to each radiograph [32]; in the case of a disagreement, the highest of the two scores was retained. In multi-rooted teeth, the root with the highest score was used as the reference. Following the assignment of a PAI score, the radiographs of each tooth were divided into two groups: absence of AP (score 1) or presence of AP (score 2–5).

The same examiners then assessed the quality of the coronal restorations according to the criteria described by Ng et al [3, 6].

### Outcome assessment

Treatment success was assessed using two outcome measures.

The primary outcome measure was “periapical healing”, including clinical and radiographic evidence of the healing of each tooth or the absence of apical periodontitis [3]. Treatment success was defined according to strict criteria as the absence of pain or clinical evidence of inflammation or swelling and by conventional radiographic measures of complete healing/continuous presence of a normal periodontal ligament space (PAI score < 2).

The secondary outcome measure was “tooth survival”. Success was achieved if the tooth was asymptomatic and considered to be functional regardless of its PAI score [33].

If a tooth had been extracted because of endodontic problems (persistent pain, swelling, or sinus or periapical radiolucent lesion), treatment was considered to have failed. Tooth extraction without any exit data regarding the postoperative periapical status excluded the tooth from further analysis for “periapical healing”. The whole tooth was considered the assessment unit.

### Statistical analysis

Continuous variables were reported as the mean ± SD, while dichotomous variables were reported as the number of cases and the percentage; qualitative ordinal variables were reported as the number of cases and the median value. Different tests were used to verify the presence of a statistically significant difference between the BIO and PCS groups; the test for the equality of proportions, *t* tests for the equality of means, and non-parametric k-sample tests for the equality of medians were applied when appropriate. The level of significance was set at 5% (*p* < 0.05); STATA version 14 (STATA Corp., TX, USA) was used to perform all statistical analyses.

## Results

The follow-up rate of this study was 82%. Of the 55 patients originally enrolled, who had 84 treated teeth, 13 patients with 15 treated teeth (2 of which were extracted without further information collection) failed to attend the follow-up appointments and were excluded from the analysis. The patients who were excluded from the statistical analysis exhibited characteristics similar to those of the patients included.

Sixty-nine teeth from 42 patients were then included in the outcome assessment (Table 1), and they were therefore distributed as follows:

**Table 1:** Descriptive data for the groups: general samples and subsamples

		All teeth (*N* = 69)			Teeth with AP (*N* = 52)		
		BIO group (*n* = 39)	PCS group (*n* = 30)	Test	BIOAP group (*n* = 28)	PCSAP group (*n* = 24)	Test
Sex	Female	27 (69.23%)	12 (40.00%)	0.0152*	20 (71.43%)	11 (45.8%)	0.0608*
	Male	12 (30.77%)	18 (60.00%)		8 (28.57%)	13 (54.2%)	
Age	Mean ***± s***t.dev	55.44 ± 15.04	56.37 ± 20.21	0.8270**	56.79 ± 16.02	52.67 ± 19.97	0.4132**
Type of treatment	Primary	13 (33.33%)	20 (66.67 %)	0.0060*	8 (28.57%)	17 (70.83%)	0.0024*
	Secondary	26 (66.67%)	10 (33.33%)		20 (71.43%)	7 (29.17%)	
Initial PAI	Mean	2.54 ± 1.43	2.70 ± 1.29	0.6292**	3.14 ± 1.24	3.13 ± 1.08	0.9563**
	Median	2	2.5	0.4760***	3	3	0.8780***
	1	11 (28.21%)	6 (20.00%)		-	-	
	2	13 (33.33%)	9 (30.00%)		13 (46.43%)	9 (37.5%)	
	3	4 (10.26%)	6 (20.00%)		4 (14.29%)	6 (25.00%)	
	4	5 (12.82%)	6 (20.00%)		5 (17.86%)	6 (25.00%)	
	5	6 (15.38%)	3 (10.00%)		6 (21.43%)	3(12.50%)	

*Test for the equality of proportions

**t tests for the equality of means

***Non-parametric K-sample test for the equality of medians

BIO group = 39 teeth in 23 patients (26.09% males and 73.91% females, average age = 53.17 years) obturated using the single-cone technique and BioRoot^TM^ RCS (Septodont).

PCS group = 30 teeth in 19 patients (47.37% males and 52.63% females, average age = 51.26 years) obturated with the warm vertical compaction technique using gutta-percha and Pulp Canal Sealer^TM^ EWT (Kerr©).

The teeth with AP from both groups were then further divided into two subsamples: BIOAP = 28 teeth in 18 patients from the BIO group, and PCSAP = 24 teeth in 16 patients from the PCS group (Table 1).

The BIO and PCS groups were homogeneous in terms of age and initial periapical status; the majority of the BIO group comprised females, while the subsamples of patients with teeth with AP were homogeneous in terms of gender. The BIO group included more secondary RCTs than the PCS group. The test used to assess whether the type of treatment was equally distributed between groups indicated that there was no significant difference and that the two groups were comparable (Table 1).

**Fig. 1:**
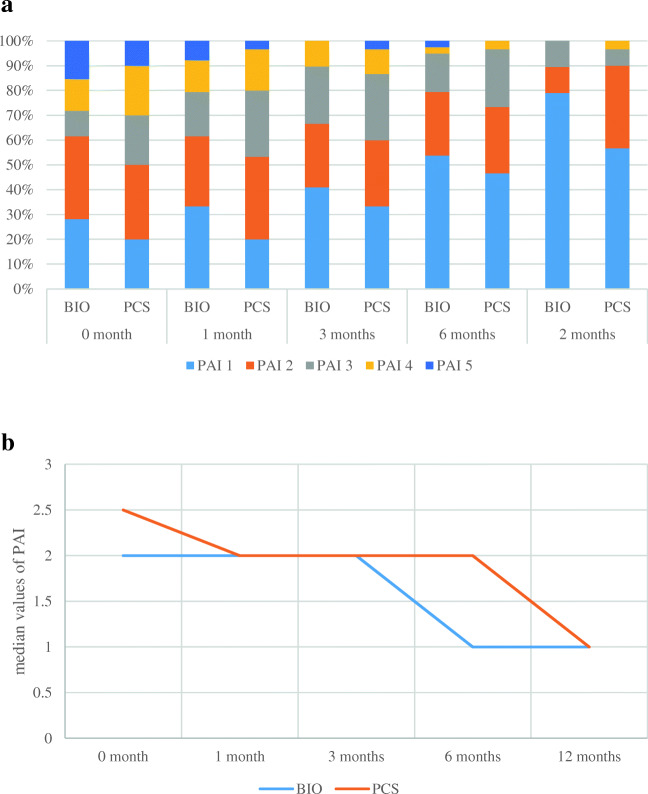
**a** PAI value distribution per treatment group at every follow-up—general samples. **b** Median values of PAI over time per treatment group—general samples

**Fig. 2:**
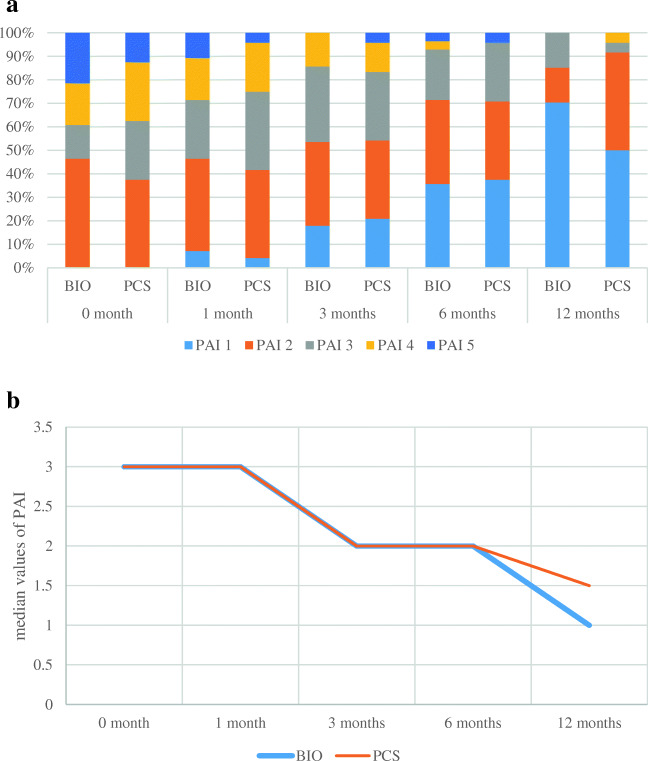
**a** PAI value distribution per treatment group at every follow-up—subsamples of teeth with AP. **b** Median values of PAI over time per treatment group—subsamples of teeth with AP

The overall survival rate of treated teeth at 12 months was 97.44% in the BIO group and 93.33% in the PCS group (*p* = 0.4074). The success rate at 12 months was higher in the BIO group than in the PCS group (76.92% versus 56.67%), although the difference was not significant (*p* = 0.0735). Similarly, the survival rates in the BIOAP and PCSAP groups were comparable at 12 months, and the healing rate was higher in the BIOAP group than in the PCSAP group (67.86% versus 50.00%), but the difference was not statistically significant (*p* = 0.1908) (Table 2). A successful treatment was found at the 12-month follow-up for all teeth without a preoperative periapical lesion. The PAIs for each treatment group and subgroup are shown in Figs. 1 and 2, respectively. Globally, all PAIs decreased over time. For all teeth with a PAI of 5 or 4 at baseline, the score was decreased at the 12-month follow-up in the BIOAP groups, while teeth with a PAI of 4 did not show a change at 12 months in the PCSAP groups (Figs. 1a and 2a).

**Table 2:** Between-group comparison in terms of the two outcomes (healing and survival) in the general samples and subsamples

		All teeth (*N* = 69)			Teeth with AP (*N* = 52)		
		BIO group (*n* = 39)	PCS group (*n* = 30)	Test	BIOAP group (*n* = 28)	PCSAP group (*n* = 24)	Test
Healed	No	9 (23.08%)	13 (43.33%)	0.0735*	9 (32.14%)	12 (50%)	0.1908*
	Yes	30 (76.92%)	17 (56.67%)		19 (67.86%)	12 (50%)	
Survival	No	1 (2.56%)	2 (6.67%)	0.4074*	1 (3.57%)	1 (4.17%)	0.9114*
	Yes	38 (97.44%)	28 (93.33%)		27 (96.43%)	23 (95.83%)	

*Test for the equality of proportions

## Discussion

The aim of endodontic filling is to finalize the treatment by sealing, as hermetically as possible, the root canal space and prevent microleakage, which may cause treatment failure [8, 9]. Endodontic obturation is traditionally performed with a GP master cone combined with a sealer, which may be further adapted to the prepared canal using a compaction technique with heat [29] or multiple accessory cones [28]. The use of a well-fitting cone in conjunction with a bioactive sealer that should enhance the sealing properties when in contact with fluids and lead to successful single-cone obturation has been recently advocated [21]. The technique is easy and fast and aims to create a biological seal [10–16, 18–20, 22, 23, 26, 27]. We designed this randomized pilot study to obtain the first insights into this technique because there was, and still is, a paucity of clinical information in the specialized literature on the use of the new bioactive cements as sealers with single-cone obturation. This article reports the 12-month follow-up of a modular project in which the outcomes of primary and secondary root canal treatments were assessed in teeth obturated with either the single-cone technique and a bioactive sealer or with continuous compaction with GP and ZOE sealer, which is considered a classic reference treatment [34].

To minimize bias, four endodontic residents performed all the treatments in the same clinical setting using a standardized instrumentation and disinfection protocol. In addition, each tooth was restored with direct composite upon the completion of RCT. The sample size was small, but the follow-up rate was good (Table 1). We have combined primary and secondary endodontic therapies to empower the statistical analysis. This choice was also supported by the literature, as the documented success rates of initial treatment and retreatment were similar as long as the teeth to be retreated did not exhibit visibly altered root canal morphology [3, 35–37].

The follow-ups were conducted at short intervals (1 month, 3 months, and then every 3 months) to obtain detailed information on the healing course of the teeth during the first year of treatment to evaluate a new material with supposedly better sealing ability (Figs. 1 and 2) [21]. This information may be useful in clinical practice, especially for teeth with extensive lesions requiring prosthetic rehabilitation. This is also the reason we intend to report the treatment outcomes progressively (at 1, 2, and 4 years).

The overall survival and success rates at 12 months were good and comparable in both treatment groups (Table 2).

All teeth without a preoperative periapical lesion did not develop signs and/or symptoms of AP at 12 months.

The degree of healing in the teeth with AP at the first-year follow-up was slightly but not significantly better in the BIOAP group than in the PCSAP group, confirming the validity of both the established and the relatively new technique. Interestingly, in the BIOAP group, all teeth with extensive lesions with an initial PAI of 5/4 showed a significant reduction in the PAI to 3/1 at 12 months (Fig. 2a). This result seems promising, since the odds of healing for AP decrease for larger lesions [3, 38]. Unfortunately, the roots were not matched in terms of the PAI in the two groups when the study was started. The potential benefits of the single-cone/bioactive sealer combination are that this technique is simple and can be implemented with a more conservative root canal preparation, and the biocompatibility of the sealer is reportedly optimal [11, 12, 39].

To the best of our knowledge, this is the first clinical trial that has compared the outcomes of the treatment of teeth obturated with a bioactive sealer and a single cone and teeth subjected to warm vertical compaction with gutta-percha and a ZOE sealer, which is a standard endodontic procedure for obturation [9]. These results are encouraging and consistent with those obtained from a recent retrospective study by Chybowski et al. [38], who reported an overall healing rate of 83.1% after an average of 30.1 months for 307 teeth that were endodontically treated or retreated with a bioactive sealer (EndoSequence Bioceramic Sealer, BC; Brasseler USA, Savannah, GA) and the single-cone technique. The better results obtained in their study are probably related to the longer follow-up time compared with that used in this initial prospective report. Unfortunately, the two investigations are not fully comparable because the tested sealers were both bioactive but different, and the authors dichotomized the outcomes as either healed or healing cases, all of which were considered “successful”, while we used strict criteria [3, 4]. Furthermore, in the other study, four endodontists performed the RCTs using different protocols in private practices, while in this study, four residents performed the RCTs in a university setting with the same protocol. These differences make the data collected for single-cone obturation with a bioactive sealer in this study even more reliable. The most interesting difference in the results of the two clinical studies is that Chybowski et al. [38] found a negative predictive value for healing for lesions > 5 mm, while we observed a fast reduction in the sizes of larger lesions at 12 months (Fig. 2a).

**Fig. 3:**
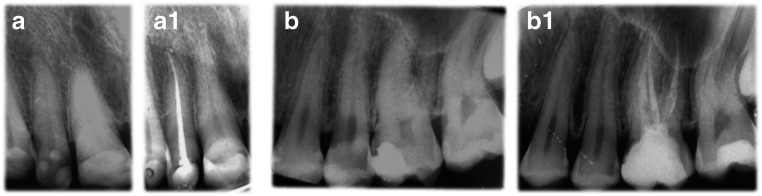
Representative case from the single-cone technique and BioRoot^TM^ RCS group, at month 0 (**a**) and 12 months follow-up (**a1**). Representative case from the warm vertical compaction of gutta-percha with zinc oxide-eugenol sealer group, at month 0 (**b**) and 12 months follow-up (**b1**)

Among the limitations of this study, the number of patients enrolled was not high, which reduces the statistical power of the research and produces extreme variability in the dental conditions, which may lead to obvious difficulties in the comparisons of the samples (Fig. 3).

## Conclusion

Based on the findings of this pilot investigation, it is possible to advance the hypothesis that the use of a bioactive sealer together with the single-cone obturation technique may represent a good filling alternative to the use of warm vertical compaction with GP and ZOE sealer. To confirm this hypothesis, the cases included in this report will need to be followed up for a longer period of time, and further trials should be performed.

## References

[1] Nair PN (1997). Apical periodontitis: a dynamic encounter between root canal infection and host response. Periodontol.

[2] Marton IJ, Kiss C (2014). Overlapping protective and destructive regulatory pathways in apical periodontitis. J Endod.

[3] Ng Y-L, Mann V, Gulabivala K (2011). A prospective study of the factors affecting outcomes of nonsurgical root canal treatment: part 1: periapical health. Int Endod J.

[4] Ng Y-L, Mann V, Gulabivala K (2011). A prospective study of the factors affecting outcomes of non-surgical root canal treatment: part 2: tooth survival. Int Endod J.

[5] Ng Y-L, Mann V, Rahbaran S, Lewsey J, Gulabivala K (2007). Outcome of primary root canal treatment: systematic review of the literature - part 1. Effects of study characteristics on probability of success. Int Endod J.

[6] Ng Y-L, Mann V, Rahbaran S, Lewsey J, Gulabivala K (2008). Outcome of primary root canal treatment: systematic review of the literature -- part 2. Influence of clinical factors. Int Endod J.

[7] Ng Y-L, Mann V, Gulabivala K (2008). Outcome of secondary root canal treatment: a systematic review of the literature. Int Endod J.

[8] (1994) Consensus report of the European Society of Endodontology on quality guidelines for endodontic treatment. Int Endod J 27:115–124. 10.1111/j.1365-2591.1994.tb00240.x 10.1111/j.1365-2591.1994.tb00240.x 7995643

[9] (2006) Quality guidelines for endodontic treatment: consensus report of the European Society of Endodontology. Int Endod J 39:921–930. 10.1111/j.1365-2591.2006.01180.x 10.1111/j.1365-2591.2006.01180.x 17180780

[10] Torabinejad M, Parirokh M, Dummer PMH (2018). Mineral trioxide aggregate and other bioactive endodontic cements: an updated overview - part II: other clinical applications and complications. Int Endod J.

[11] Parirokh M, Torabinejad M (2010). Mineral trioxide aggregate: a comprehensive literature review--part I: chemical, physical, and antibacterial properties. J Endod.

[12] Torabinejad M, Parirokh M (2010). Mineral trioxide aggregate: a comprehensive literature review--part II: leakage and biocompatibility investigations. J Endod.

[13] Gomes-Filho JE, Watanabe S, Lodi CS, Cintra LTA, Nery MJ, Filho JAO, Dezan E, Bernabé PFE (2012). Rat tissue reaction to MTA FILLAPEX(R). Dent Traumatol.

[14] Salles LP, Gomes-Cornelio AL, Guimaraes FC (2012). Mineral trioxide aggregate-based endodontic sealer stimulates hydroxyapatite nucleation in human osteoblast-like cell culture. J Endod.

[15] Lee B-N, Lee K-N, Koh J-T, Min KS, Chang HS, Hwang IN, Hwang YC, Oh WM (2014). Effects of 3 endodontic bioactive cements on osteogenic differentiation in mesenchymal stem cells. J Endod.

[16] Zhou H, Du T, Shen Y (2015). In vitro cytotoxicity of calcium silicate-containing endodontic sealers. J Endod.

[17] Zhang W, Li Z, Peng B (2010). Effects of iRoot SP on mineralization-related genes expression in MG63 cells. J Endod.

[18] Loushine BA, Bryan TE, Looney SW, Gillen BM, Loushine RJ, Weller RN, Pashley DH, Tay FR (2011). Setting properties and cytotoxicity evaluation of a premixed bioceramic root canal sealer. J Endod.

[19] Borges RP, Sousa-Neto MD, Versiani MA, Rached-Júnior FA, de-Deus G, Miranda CES, Pécora JD (2012). Changes in the surface of four calcium silicate-containing endodontic materials and an epoxy resin-based sealer after a solubility test. Int Endod J.

[20] Faria-Junior NB, Tanomaru-Filho M, Berbert FLCV, Guerreiro-Tanomaru JM (2013). Antibiofilm activity, pH and solubility of endodontic sealers. Int Endod J.

[21] Wang Z (2015). Bioceramic materials in endodontics. Endod Top.

[22] Assmann E, Bottcher DE, Hoppe CB (2015). Evaluation of bone tissue response to a sealer containing mineral trioxide aggregate. J Endod.

[23] Zhang W, Li Z, Peng B (2010). Ex vivo cytotoxicity of a new calcium silicate-based canal filling material. Int Endod J.

[24] Zhang H, Shen Y, Ruse ND, Haapasalo M (2009). Antibacterial activity of endodontic sealers by modified direct contact test against Enterococcus faecalis. J Endod.

[25] Wang Z, Shen Y, Haapasalo M (2014). Dentin extends the antibacterial effect of endodontic sealers against Enterococcus faecalis biofilms. J Endod.

[26] Camps J, Jeanneau C, El Ayachi I (2015). Bioactivity of a calcium silicate-based endodontic cement (BioRoot RCS): interactions with human periodontal ligament cells in vitro. J Endod.

[27] Camilleri J (2015). Sealers and warm gutta-percha obturation techniques. J Endod.

[28] Berman L, Hargreaves KM (2015) Cohen’s pathways of the pulp expert consult, 11th edn. Elsevier

[29] Buchanan LS (1994). The continuous wave of condensation technique: a convergence of conceptual and procedural advances in obturation. Dent Today.

[30] Bose R, Nummikoski P, Hargreaves K (2009). A retrospective evaluation of radiographic outcomes in immature teeth with necrotic root canal systems treated with regenerative endodontic procedures. J Endod.

[31] Landis JR, Koch GG (1977). The measurement of observer agreement for categorical data. Biometrics.

[32] Orstavik D, Kerekes K, Eriksen HM (1986). The periapical index: a scoring system for radiographic assessment of apical periodontitis. Endod Dent Traumatol.

[33] Friedman S, Mor C (2004). The success of endodontic therapy--healing and functionality. J Calif Dent Assoc.

[34] Castellucci A, Rotstein I, Ingle IJ (2019). Obturation of the radicular spaces. Ingle’s Endodontics 7.

[35] Friedman S, Abitbol S, Lawrence HP (2003). Treatment outcome in endodontics: the Toronto study. Phase 1: initial treatment. J Endod.

[36] Farzaneh M, Abitbol S, Friedman S (2004). Treatment outcome in endodontics: the Toronto study. Phases I and II: orthograde retreatment. J Endod.

[37] Gorni FGM, Gagliani MM (2004). The outcome of endodontic retreatment: a 2-yr follow-up. J Endod.

[38] Chybowski EA, Glickman GN, Patel Y, Fleury A, Solomon E, He J (2018). Clinical outcome of non-surgical root canal treatment using a single-cone technique with endosequence bioceramic sealer: a retrospective analysis. J Endod.

[39] Ghoneim AG, Lutfy RA, Sabet NE, Fayyad DM (2011). Resistance to fracture of roots obturated with novel canal-filling systems. J Endod.

