# Correction to: A 12-month follow-up of primary and secondary root canal treatment in teeth obturated with a hydraulic sealer

**DOI:** 10.1007/s00784-021-04026-z

**Published:** 2021-06-18

**Authors:** Giulia Bardini, Laura Casula, Emanuele Ambu, Davide Musu, Montse Mercadè, Elisabetta Cotti

**Affiliations:** 1 Department of Conservative Dentistry and Endodontics, University of Cagliari, Cagliari, Italy; 2 Department of Medicine and Public Health, University of Cagliari, Cagliari, Italy; 3 Department of Dentistry, University of Barcelona, Barcelona, Spain

**Correction to: Clinical Oral Investigations**

10.1007/s00784-020-03590-0

The article “A 12-month follow-up of primary and secondary root canal treatment in teeth obturated with a hydraulic sealer”, written by Giulia Bardini, Laura Casula, Emanuele Ambu, Davide Musu, Montse Mercadè and Elisabetta Cotti, was originally published Online First without Open Access. After publication in volume 25, issue 5, page 2757–2764 the author decided to opt for Open Choice and to make the article an Open Access publication. Therefore, the copyright of the article has been changed to © The Author(s) 2021 and the article is forthwith distributed under the terms of the Creative Commons Attribution 4.0 International License, which permits use, sharing, adaptation, distribution and reproduction in any medium or format, as long as you give appropriate credit to the original author(s) and the source, provide a link to the Creative Commons license, and indicate if changes were made. The images or other third party material in this article are included in the article’s Creative Commons license, unless indicated otherwise in a credit line to the material. If material is not included in the article’s Creative Commons license and your intended use is not permitted by statutory regulation or exceeds the permitted use, you will need to obtain permission directly from the copyright holder. To view a copy of this license, visit http://creativecommons.org/licenses/by/4.0.

The original article has been corrected.

